# Development of anoikis-related genes signature to predict the prognosis in gastric cancer patients

**DOI:** 10.3389/fonc.2022.1096608

**Published:** 2023-01-12

**Authors:** Jie Cao, Kai Hong, Yuepeng Cao, Kenan Cen, Yifeng Mai, Ying Dai, Guifang Ouyang, Qitian Mu, Yangyang Guo

**Affiliations:** ^1^ Laboratory of Stem Cell Transplantation, Ningbo First Hospital, Ningbo, China; ^2^ Department of General Surgery, Ningbo First Hospital, Ningbo, China; ^3^ The Affiliated Hospital of Medical School of Ningbo University, Ningbo, China; ^4^ Department of Hematology, Ningbo First Hospital, Ningbo, China

**Keywords:** anoikis, gastric cancer, immune microenvironment, immunotherapy, prognosis

## Abstract

**Background:**

It is well known that the prognosis of Gastric cancer (GC) patient is affected by many factors. However, the latent impact of anoikis on the prognosis of GC patients is insufficient understood.

**Methods:**

According to the Cancer Genome Atlas (TCGA) database, we elected discrepantly expressed anoikis-related genes (ARGs). Univariate cox and the least absolute shrinkage and selection operator (lasso) analysis were applied to build the ARGs signature. The prognostic effect of the ARGs signature was also evaluated. A series of algorithms were performed to evaluate the discrepancies in the immune microenvironment. Moreover, the correlation between drug sensitivity and ARGs signature was analyzed. We also performed Real-Time Polymerase Chain Reaction (RT-PCR) to probe the signature.

**Results:**

The ARGs signature of 9 genes was constructed, which was apparently interrelated with the prognosis. The nomogram was established by combining the ARGs signature with clinicopathological characteristics. We found that the predictive power was noteworthily superior to other individual predictors. The immune microenvironment analysis indicated that ESTIMATEscore, ImmuneScores, StromalScores, tumor immune dysfunction and exclusion (TIDE) score were lower in the low-risk group, while immunophenoscore (IPS) was on the contrary. The infiltrated immune cells and immune checkpoint (ICP) expression levels were significantly different between the two groups. Furthermore, nine drugs were positively associated with the ARGs signature score. The results of RT-PCR analysis were consistent with our previous differential expression analysis.

**Conclusion:**

The developed ARGs signature could act as the biomarker and provide a momentous reference for Individual therapy of GC patients.

## Introduction

As an extremely heterogeneous and highly aggressive malignant tumor, gastric cancer (GC) has a high morbidity and mortality rates worldwide. The Global Cancer Report 2020 said there were more than nearly 760,000 dead patients and 1,000,000 new patients globally each year (1). In the past few decades, surgery, examination techniques and adjuvant therapy had made significant progress, but the prognosis and outcome of GC patients was still very poor, particularly for those with advanced GC patient whose five-year overall survival was less than 20% (2). Although GC patients had semblable grading of tumor and identical pathological staging, their survival outcomes could be completely disparate based on the different genetic features. Therefore, it is extremely vital to identify efficient and reliable biomarkers and clinical therapy methods to facilitate the survival and remedy of patients with GC.

Recently, several reports had confirmed that tumor cells could carry extracellular matrix (ECM) in the process of metastasis (3, 4). During this process, the anoikis was triggered when cancer cells detangle from the ECM. So, the anoikis, resulting from the separation of tumor cells from the ECM, serves as a peculiar modality of apoptosis (5). The anoikis was initially discovered in endothelial and epithelial cells, which was acted as a physiological procedure relevant to tissue homeostasis and upgrowth (6). Apoptosis plays a momentous role in safeguarding the organisms by preventing already isolated cells from reconnecting to else substrates for aberrant proliferation. Nevertheless, the failure of the anoikis prognosis process to proceed properly may lead to adherent cells proliferating in the ECM distinct from *in situ* or surviving in suspension (7). The obstruction of initiation of the apoptosis program promotes tumorigenesis and the progression of distal tumor metastasis.

Several studies have indicated that the genes associated with anoikis played a central role in cancer progression and tumor metastasis, such as endometrial carcinoma (EC) (8), lung cancer (LC) (9), breast carcinoma (BC) (10) and GC (11). For instance, a study showed that over-expression of FAIM2 was associated with adverse clinical prognosis in LC, and knockout FAIM2 could inhibit anoikis resistance and cancer cell viability (12). As an original prognostic factor, KLF5 has been demonstrated to modulate anoikis resistance and cell proliferation of colorectal cancer (CRC) patients. In the HEC-1A cells line, L1CAM could promote the upgrowth of tumor initiating cells to accelerate epithelial mesenchymal transformation (EMT), thereby facilitating the resistance of anoikis and affecting the tumor progression of GC patients. This suggests that anoikis-related genes may serve as the novel tumor markers. Therefore, it is very necessary to conduct extensive research on anoikis-related genes in GC patients. Although these studies have confirmed the association of anoikis with cancer progression and tumor metastasis, ARGs-based prognostic models in GC have been rarely explored (13, 14).

In this research, we obtained the expression data of anoikis-related genes (ARGs) and clinic information from the public database. The ARGs signature has been established by the univariate cox analysis and the least absolute shrinkage and selection operator (lasso) analysis. In addition, the prognostic role of the ARGs signature in the clinic was explored, which provided the basis for individualized therapy of GC patients.

## Materials and methods

### Data processing

The clinic data and gene expression information of GC specimens were extracted from the Cancer Genome Atlas (TCGA) database, which included 371 tumor specimen specimens and 32 normal specimens. The RNA-sequencing expression data were normalized with the FPKM method. The GSE84437 dataset was extracted from the Gene Expression Omnibus (GEO) database, which included 433 tumor specimens with available clinic information. The data were standardized to eliminate batch impacts. The data from TCGA were acted as the training dataset and the data from GEO were acted as the testing dataset. The ARGs were gained from GeneCards database. A relevance score >0.4 was served as the screening condition (**Supplementary Table 1**).

### Development of ARGs signature

According to the ARGs, the differentially expressed genes (DEGs) between normal specimens and tumor specimens were selected in the training dataset (|log FC| > 1, p value < 0.05). Subsequently, univariate cox analysis was employed to further select prognostic related genes. The lasso analysis was applied to eliminate overfitting genes. Finally, a novel ARGs signature was developed by lasso analysis. A ARGs-based risk score was calculated by the following process: risk score= expr-gene1 × coefficient1 + expr-gene2 × coefficient2 +… + expr-genen × coefficientn. Based on the ARGs signature score, we choose the median as a cut-off value.

### Assessment of the ARGs signature prognosis value

To assess the prognosis value of ARGs signature, the score of GC patients in the training dataset was obtained. According to the cutoff value, each GC patient was split into low-risk class or high-risk class. The Kaplan-Meier (K-M) analysis was performed to forecast the overall survival (OS) of GC patients in the low-risk or high-risk classes. The receiver operator characteristic (ROC) curve was employed to prove the specificity and sensitivity of ARGs signature. To detect whether the ARGs signature acted as an independent marker of the GC prognosis, the univariate/multivariate regression analysis proceeded for the clinicopathological characteristics and ARGs signature. Meanwhile, the above content was verified in the testing dataset. Moreover, a nomogram was established to predict the outcome of patients, on the basis of the clinicopathological characteristics and ARGs signature in the TCGA cohort. We employed calibrated graph to appraisal the dependability of the ARGs signature. The ROC analysis was employed to compare the prognosis value of single clinicopathological characteristics, ARGs signature and nomogram model.

### Exploration of immune microenvironment

Immune cell abundance (ImmuneScores) and stromal cell abundance (StromalScores) were evaluated by the ESTIMATE. The XCELL, QUANTISEQ, TIMER, MCPCOUNTER, EPIC, CIBERSORT and CIBERSORT-ABS were performed to explore the difference in the infiltration levels of immune cells between low and high risk classes. The single sample gene set enrichment analysis (ssGSEA) was applied to study the differences between two groups in immune function and immune cell infiltration. The immune checkpoint (ICP) expression levels were also calculated. Microsatellite instability (MSI) and tumor mutational burden (TMB) acted as vital factors. The difference between low and high risk classes was also probed. To explore the underlying immunotherapy response of patients, immunophenoscore (IPS) and tumor immune dysfunction and exclusion (TIDE) score were examined.

### Pathway analysis of the ARGs signature

The differential genes between low and high risk classes were screened in the TCGA cohort. The underlying pathway analysis associated with differential genes was enriched through the gene ontology (GO) analysis and kyoto encyclopedia of genes and genomes (KEGG). The GO analysis was divided into three categories, including Molecular Function, Biological Process and Cellular Component. The gene set variation analysis (GSVA) was performed to probe the difference of the potential biological function between the high-risk patients and low-risk patients. The adj.p-value below 0.05 was statistically significant.

### Drug sensitivity analysis

We researched the forecasting ability of ARGs signature for chemotherapy and targeted therapy drugs. The pRRophetic method was applied to compute the half-maximal inhibitory concentration (IC50). The IC50 denoted the availability of a drug in controlling a specific biochemical or biological process.

### Real-Time Polymerase Chain Reaction (RT-PCR)

Total RNA was obtained from normal stomach cell line and GC cancer cell line using TRIzol reagent (Invitrogen). Reverse transcriptase reaction was performed using PrimeScript™RT kit (TaKaRa). β-actin was used to normalize mRNAs expression levels of genes. Normalized CT values were used to calculate the fold difference of each group. Primer sequences were shown in **Supplementary Table 2**.

## Results

### DEGs in GC and normal tissues

**Figure 1:**
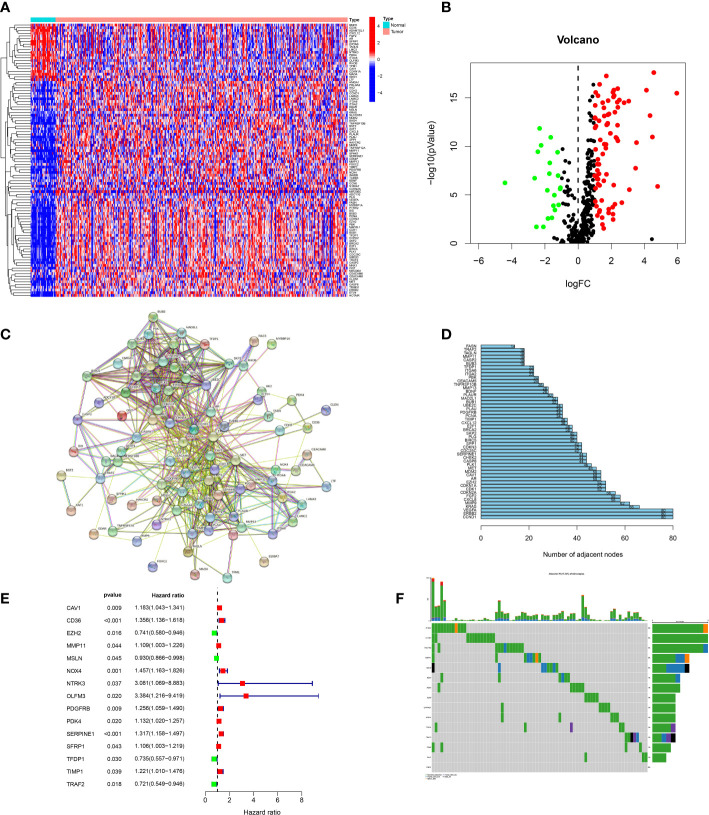
Screening of candidate genes. **(A)** Heatmap of DEGs between tumor specimens and normal specimens. **(B)** A volcanic plot of DEGs between tumor specimens and normal specimens. **(C)** The PPI network of DEGs. **(D)** The number of nodes of hub genes in the PPI network. **(E)** Forest plot of genes associated with prognosis. **(F)** Mutations of prognostic genes.

We obtained 434 ARGs from GeneCards database. The expression levels of 434 ARGs in the normal and GC specimens were acquired from the TCGA dataset. The differential analysis found 102 DEGs between normal and GC tissues, including 21 down-regulated and 81 upregulated genes (**Figures 1A, B**). Then, the protein–protein interaction (PPI) analysis of 102 DEGs was built to show the pivotal nodes (**Figure 1C**). We found that CCND1, ERBB2 and VEGFA were the important genes (**Figure 1D**).

### Construction of the ARGs signature

**Figure 2:**
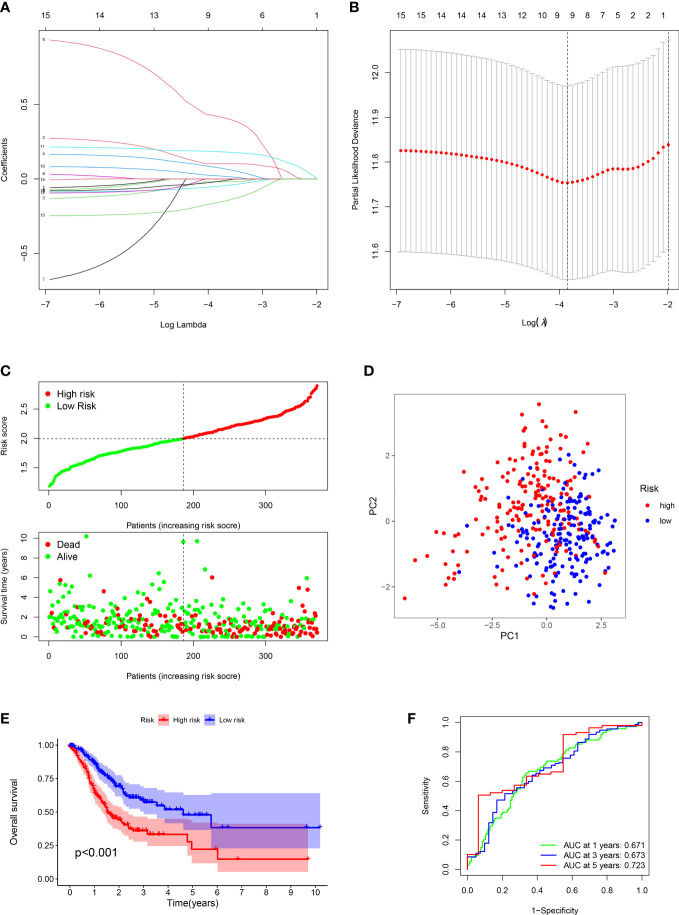
Construction of prognostic signature and evaluation of prognostic effect in TCGA dataset. **(A, B)** The LASSO analysis of the candidate genes. **(C)** Distribution of risk scores for each GC sample and survival time and status for each GC sample based on risk scores. **(D)** The PCA analysis of GC patients between high and low risk groups. **(E)** Survival differences between GC patients between high and low risk groups. **(F)** ROC analysis of GC patients.

In order to build a more precise ARGs signature, univariate regression analysis was used to gain 15 prognosis-related genes from 102 DEGs (**Figure 1E**). The genes mutation analysis of 15 prognosis-related genes showed that 17.32% of patients had genes mutation (**Figure 1F**). The three genes (NTRK3, OLFM3, PDGFRB) mutation rate was the commonest. Then, we performed the lasso analysis to remove the overfitting genes of the 15 candidate genes. Ultimately, the ARGs signature of 9 genes (CD36, EZH2, MMP11, MSLN, OLFM3, PDK4, SERPINE1, TFDP1, TRAF2) was constructed by the multivariate regression analysis. The ARGs-based risk score was calculated by the following process: risk score= (CD36 × (0.1017) + (EZH2 × (-0.019) + (MMP11 × (0.077) + (MSLN × (-0.051) + (OLFM3 × (0.4223) + (PDK4 × (0.0214) + (SERPINE1 × (0.1833) + (TFDP1 × (-0.0189) + (TRAF2× (-0.1609) (**Figures 2A, B**).

### Assessment and validation of ARGs signature

For each GC patient, the risk score was computed by the above formula in the training dataset. All patients were divided into high-risk or low-risk groups (**Figure 2C**). Principal Component Analysis (PCA) indicated that low-risk and high-risk groups had a clear distinction (**Figure 2D**). The K-M analysis manifested that the survival outcome of high-risk class was worse than the low-risk class (P < 0.001) (**Figure 2E**). The ROC plot was performed to appraisal the ARGs signature, which manifested that the AUC of 1, 3 and 5 years were 0.671, 0.673, 0.723, respectively (**Figure 2F**).

**Figure 3:**
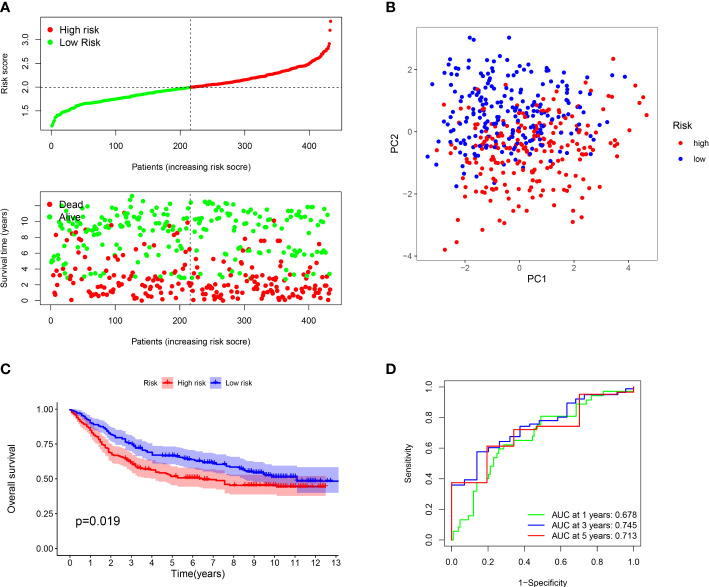
Evaluation of prognostic effect in GEO dataset. **(A)** Distribution of risk scores for each GC sample and survival time and status for each GC sample based on risk scores. **(B)** The PCA analysis of GC patients between high and low risk groups. **(C)** Survival differences between GC patients between high and low risk groups. **(D)** ROC analysis of GC patients.

**Figure 4:**
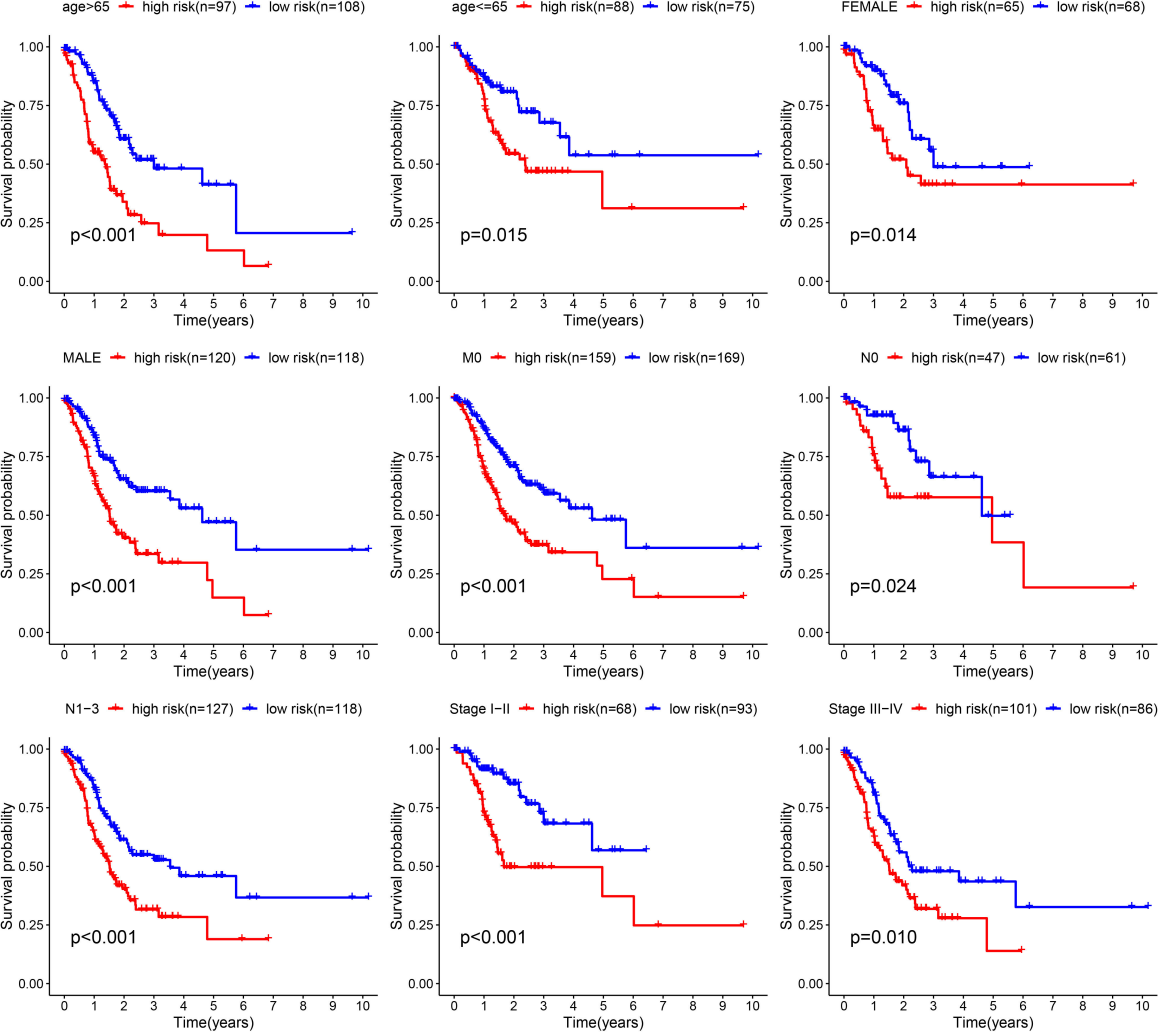
Validation of the prognostic value of ARGs signature in different clinical characteristics groups.

Furthermore, we verified the above results in the testing dataset. All GC patients were also divided into low-risk or high-risk groups (**Figure 3A**). PCA also demonstrated that two groups were obviously different (**Figure 3B**). The K-M analysis also disclosed that the high risk patients had the worse prognosis than the low risk patients (P=0.019) (**Figure 3C**). The AUC values of the ROC curve of 1, 3 and 5 years were 0.678, 0.745 and 0.713, respectively (**Figure 3D**). Moreover, we validated the prognosis prediction capacity of the ARGs signature in various clinical information in the TCGA dataset. The study indicated that the ARGs signature could forecast the outcome of patients in age <= 65 (P =0.015), age > 65 (P < 0.001), female (P =0.014), male (P < 0.001), N0 (P < 0.024), N1-3 (P < 0.001), M0 (P < 0.001), stage I-II(P < 0.001), stage III-IV(P < 0.010) (**Figure 4**).

**Figure 5:**
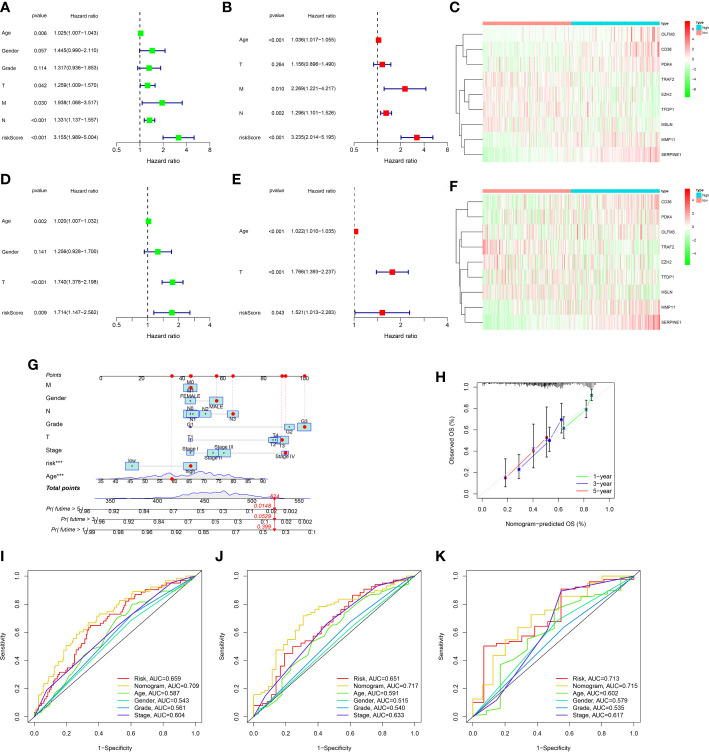
Construction and assessment of nomogram. **(A)** Univariate Cox regression analysis of the ARGs signature and clinical characteristics in the TCGA dataset. **(B)** Multivariate Cox regression analysis of the ARGs signature and clinical characteristics in the TCGA dataset. **(C)** The gene expression levels of ARGs signature in the high and low risk groups of the TCGA dataset. **(D)** Univariate Cox regression analysis of the ARGs signature and clinical characteristics in the GEO dataset. **(E)** Multivariate Cox regression analysis of the ARGs signature and clinical characteristics in the GEO dataset. **(F)** The gene expression levels of ARGs signature in the high and low risk groups of the GEO dataset. **(G)** The prediction of nomogram in the TCGA dataset. **(H)** Calibration plots for the nomogram. **(I–K)** Multifactor ROC curve for 1 year, 3 years and 5 years.

To further explore the prognosis prediction ability of the ARGs signature and clinicopathological characteristics, univariate/multivariate cox methods were performed in the TCGA dataset. The univariate cox method indicated that risk score, age, T, M, N and could influence patient prognosis (**Figure 5A**). As disclosed with the multivariate regression methods, score still affected the prognosis, manifesting the ARGs signature acted as an independent prognosis marker (**Figure 5B**). The expression levels of nine genes in ARGs signature in the low-risk and high-risk classes were displayed in the heat-map (**Figure 5C**). In addition, univariate/multivariate regression were also proceeded in the GSE84437 dataset. The univariate regression indicated that age, T and risk score could influence patient prognosis (**Figure 5D**). The multivariate regression revealed that the ARGs signature acted as an independent biomarker (**Figure 5E**). The heat-map displayed the nine genes expression levels in the low-risk and high-risk groups (**Figure 5F**). The above study proved that the ARGs signature we established could credibly predict the outcome of GC patients.

### Development of the nomogram

In order to ulteriorly exploit the prognosis effect of the ARGs signature, a novel nomogram was constructed with the ARGs signature and clinic factors in the TCGA dataset (**Figure 5G**). The calibration curves indicated that this novel nomogram had a high value for prognostic prediction (**Figure 5H**). In addition, the ROC plot was applied to compare the prognosis forecasting value of the nomogram and other single factors (age, gender, grade, stage and risk score). For 1-year survival times, the AUC value was 0.709 (nomogram), 0.659(risk score), 0.587(age), 0.543(gender) and 0.561(grade), 0.604(stage), respectively (**Figure 5I**). For 3-year survival times, the AUC value was 0.717 (nomogram), 0.651(risk score), 0.591(age), 0.515(gender) and 0.540(grade), 0.633(stage), respectively (**Figure 5J**). For 5-year survival times, the AUC value was 0.715 (nomogram), 0.713(risk score), 0.602(age), 0.579(gender) and 0.535 (grade), 0.617(stage), respectively (**Figure 5K**). These results revealed that this novel nomogram could act as the admirable prognosis prediction model.

### Analysis of tumor immune microenvironment (TIM)

**Figure 6:**
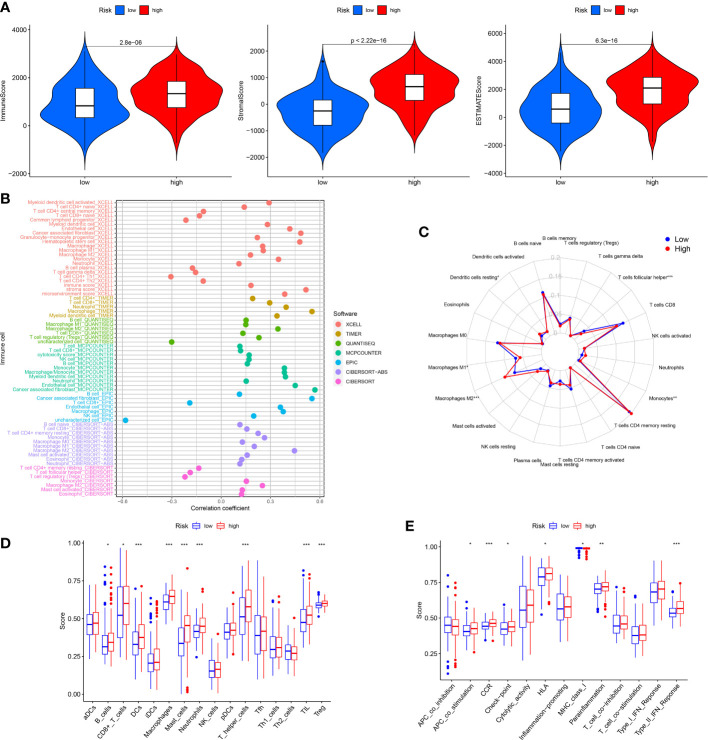
Analysis of immune conditions of high and low risk groups. **(A)** Differences of immune microenvironment scores between the two groups. **(B)** The analysis of differences in immune cell infiltration between the two groups with Multiple algorithms. **(C)** The analysis of differences in immune cell infiltration between the two groups with the CIBERSORT algorithm. **(D)** The analysis of differences in immune cell infiltration between the two groups with ssGSEA. **(E)** The differences of immune functions between the two groups with ssGSEA. *P < 0.05; **P < 0.01; ***P < 0.001.

The TIM served as a crucial indicator of the biological behavior of the tumor. In the TCGA cohort, we explored the difference of TIM between low and high risk patients. The ESTIMATE analysis revealed that the ImuneScores, StromalScores and ESTIMATEScores were all lower in the low risk patients than the high risk patients (**Figure 6A**). Moreover, the distinctions of immune cells infiltration between low and high risk patients were also investigated through XCELL, QUANTISEQ, TIMER, MCPCOUNTER, CIBERSORT-ABS, EPIC and CIBERSORT. The results showed that the levels of most immune cells were elevated in the high risk patients (**Figure 6B**). According to the CIBERSORT, for each GC patient in the low and the high risk group, we assessed the relative scale of the 22 cells of immune infiltration. The **Figure 6C** displayed the 22 cells of immune infiltration in the low-risk patients and high-risk patients. T cells follicular helper, Macrophages M1, Monocytes, Macrophages M2 and Dendritic cells resting were markedly disparate in the low risk patients and high risk patients. Furthermore, ssGSEA analysis found that the B cells, Dendritic Cells (DCs), CD8+ T cells, Macrophages, Mast cells, Neutrophils, Tumor infiltrating lymphocyte (TIL), and T helper cells T cells regulatory (Treg) infiltrated less in the low risk patients than the high risk patients (**Figure 6D**). The relevant immunologic function of CCR, APC-co-stimulation, HLA, check-point, Parainflammation and Type-II-IFN-Response were improved in the high risk patients. However, the immunologic function of MHC-class-I was improved in the low risk patients (**Figure 6E**).

**Figure 7:**
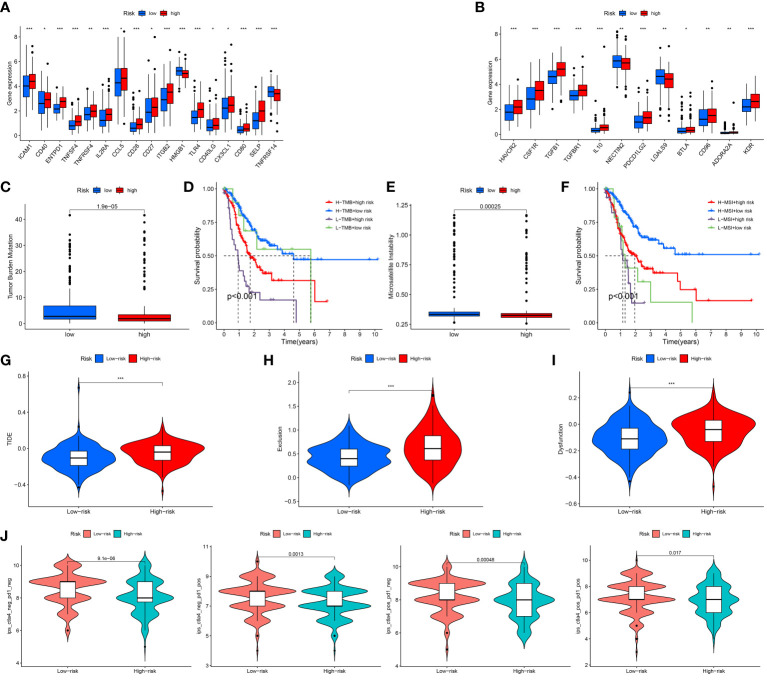
Assessment of Immunotherapy response of high and low risk groups. **(A, B)** The ICP gene expression levels in different groups. **(C)** The TMB scores in different groups. **(D)** Survival differences among patients with different TMB scores combined with different risk scores. **(E)** The MSI scores in different groups. **(F)** Survival differences among patients with different MSI scores combined with different risk scores. **(G–I)** The TIDE scores in different groups. **(J)** The IPS scores in different groups. *P < 0.05; **P < 0.01; ***P < 0.001.

As we all know, the ICP genes are vital in adjusting TIM. So, we compared the different ICP genes expression levels of in the high risk patients and the low risk patients. The result demonstrated that most of the ICP genes tended to be high-expressed in the high-risk patients (**Figures 7A, B**). To investigate the value of ARGs signature in the immune response and immune mechanism of the TIM, the TMB and MSI were analyzed. We discovered that patients with lower risk scores had higher TMB (**Figure 7C**). The K-M analysis indicated that GC patients with higher TMB and lower risk scores had a better prognosis. On the contrary, GC patients with lower TMB and higher risk scores had a worse prognosis (**Figure 7D**). Meanwhile, the GC patients with lower risk scores had higher MSI (**Figure 7E**). The same is that the survival probability was higher in the GC patients with higher MSI and lower risk scores. The survival probability was lower on the opposite (**Figure 7F**).

Under normal conditions, a higher TIDE score and lower IPS predict worse immunological therapy response. We detected the TIDE, exclusion and dysfunction score in the low and high risk classes. The results revealed that all three scores in the high-risk class were higher than those in the low-risk class (**Figures 7G–I**). In addition, the GC patients in the low-risk class had higher IPS than the GC patients in the high-risk class (**Figure 7J**). The above research indicated that the patients with the high risk scores might have a worse response to immunological therapy.

### Functional evaluation of the ARGs signature

**Figure 8:**
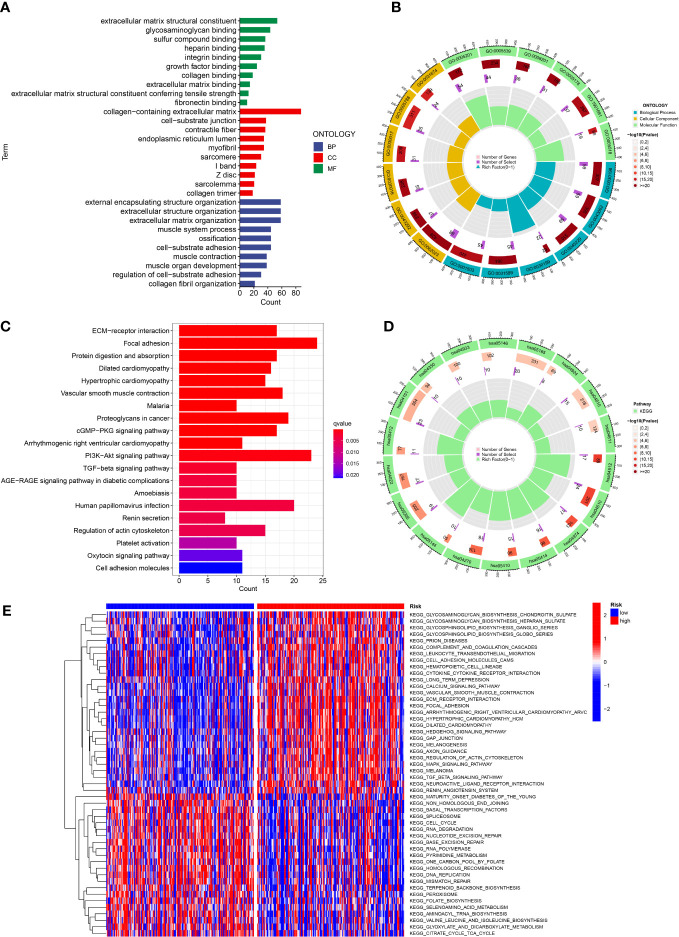
Function analysis. **(A, B)** GO analysis of differential genes between high and low risk groups. **(C, D)** KEGG analysis of differential genes between high and low risk groups. **(E)** GSVA enrichment analysis in high and low risk groups.

In order to investigate the latent function of the differential genes, the GO analysis and KEGG analysis were performed in the TCGA dataset. The GO revealed that the differential genes were mostly gathered in the extracellular matrix structural constituent, external encapsulating structure organization, collagen-containing extracellular matrix, extracellular structure organization and extracellular matrix organization (**Figures 8A, B**). The KEGG result revealed that the differential genes were mainly enriched in PI3K-Akt signaling pathway, focal adhesion and human papillomavirus infection (**Figures 8C, D**). In addition, the GSVA indicated that many pathways were apparently altered between the high and low-risk classes. For example, cell cycle, RNA degradation and RNA polymerase were apparently upregulated in the low risk GC patients (**Figure 8E**).

### The correlation analysis of drug sensitivity and risk score

**Figure 9:**
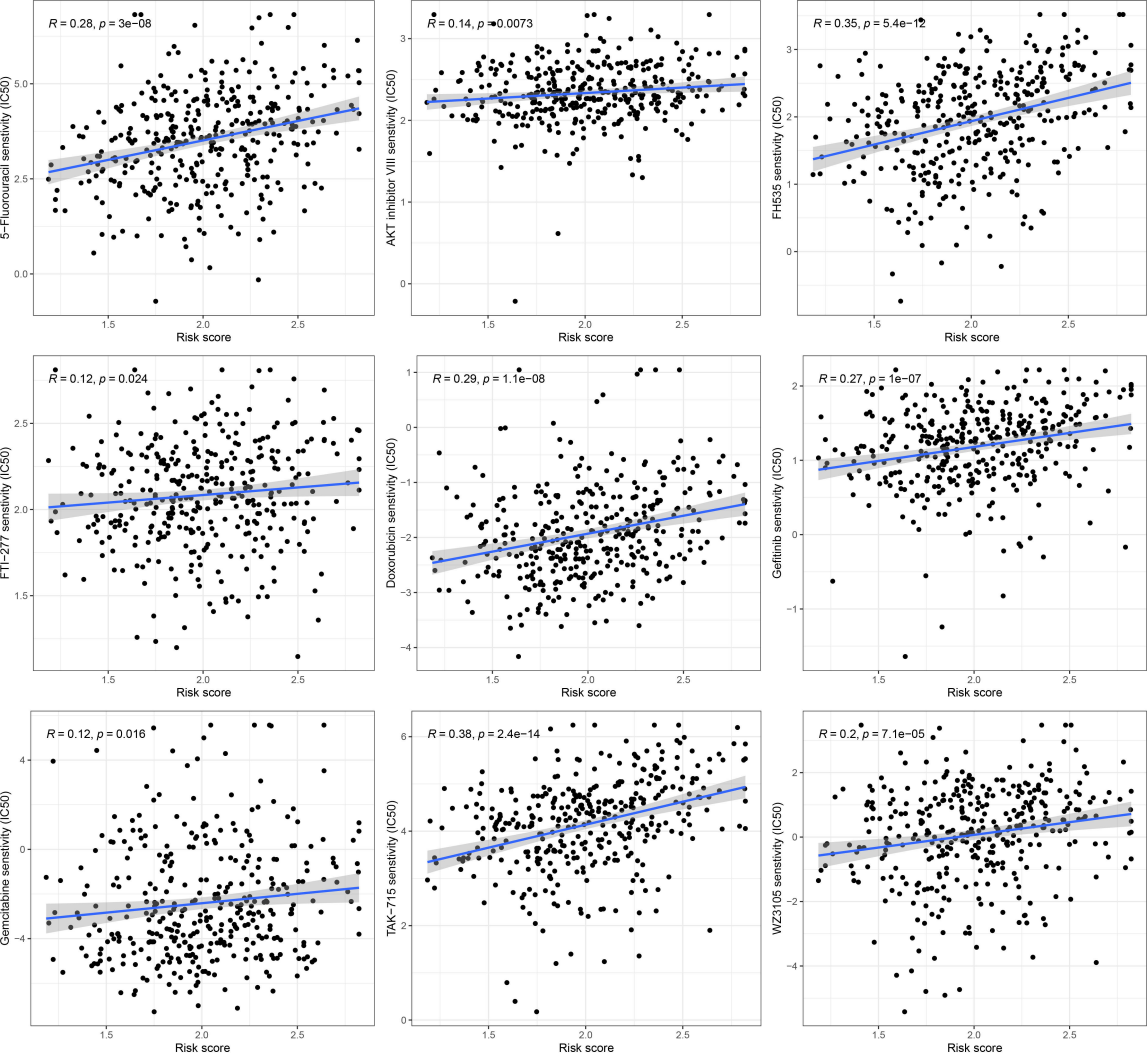
Drug sensitivity analysis in high and low risk groups.

To further explore the discrepancy of potential drug resistance between the high-risk and low-risk GC patients, we analyzed the correlation between the score and IC50 values of the targeted therapies and chemical therapies drugs in the GC patients. The IC50 value of 9 drugs (5-Fluorouracil, AKT inhibitor VIII, FH535, FTI-277, Doxorubicin, Gefitinib, Gemcitabine, TAK-715, WZ3105) was remarkably positively related with the scores of patients (**Figure 9**). This suggested that these drugs might not be effective for high-risk GC patients.

### Validation of the genes of the ARGs signature

**Figure 10:**
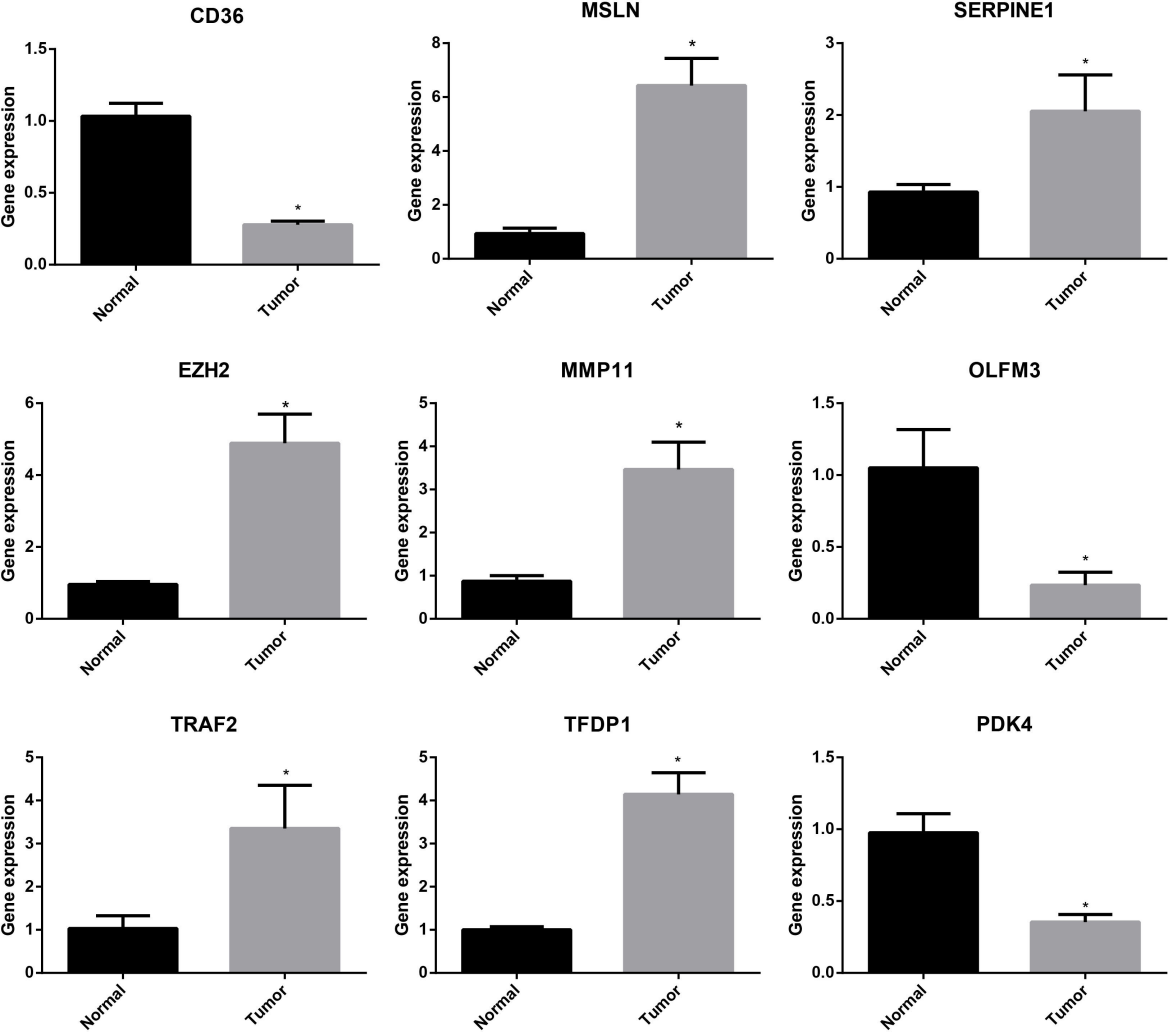
RT-PCR analysis of the genes of the ARGs signature. *P < 0.05.

To further study the expression of signature-related genes, normal cell line (GES-1) and GC cell line (HGC-27) were applied to validate the expression levels of these nine genes. The RT-PCR analysis showed that the results were consistent with our previous analysis of difference expressions. We found that 6 genes were highly expressed in GC cell line, while 3 were low expressed (**Figure 10**). These results suggested that these genes might serve as vital biomarkers for GC.

## Discussion

GC is a momentous cause of tumor-related death. Although the GC has improved in recent years, its prognosis is still poor due to tumor heterogeneity, limited treatment, and low early diagnosis rate (15). Recently, with the application of the sequencing techniques, many studies have shown that driving genes mutations and molecular pathologic subtyping affected the prognosis of cancer. However, the underlying mechanism of GC progression remains unclear. Moreover, due to the lack of novelty and rich validation, the existing models have not been widely accepted. Therefore, it is pressing to develop reliable prognostic markers to enhance prognostic prediction in GC patients. As a peculiar death modality of the apoptotic cell, anoikis has been reported to modulate the biological behaviors of diversified tumors. For instance, through the activation of the Src/FAK pathway, IQGAP1 could restrain anoikis and promote cell viability (16). Studies have proved that CCN2 could inhibit the progression of LC through the DAPK-associated anoikis pathway (17). Therefore, ARGs are potential prognostic biomarkers and treatment target points for various tumors.

In our study, we downloaded the GC-related datasets of TCGA (training group) and GEO (validation group). According to the TCGA-STAD dataset, we selected 102 differentially expressed ARGs. The univariate regression and lasso were proceeded to construct the ARGs signature, which contained nine genes. The prognosis effect of ARGs signature was evaluated in TCGA and GEO cohorts. To extend the functionality of the ARGs signature, we generated a novel nomogram that included the clinicopathological features and risk scores of the GC patient. The calibration curves proved that our nomogram had a good linear fitting for prognostic prediction.

The ARGs signature we constructed was obvious correlated with the outcome of patients with GC. The ARGs signature consists of the following 9 ARGs. Many of these ARGs have been shown to be strongly associated with cancer. For example, a study has confirmed that CD36 could mediate FA-induced GC metastasis through the AKT/GSK-3β/β-catenin pathway (18). The SNHG1 adjusted the transfer of CRC cells to affect the prognosis of patients (19). The study has indicated that MMP11 accelerated the progress of BC by suppressing the retrogradation of Smad2. The gene of MMP11 was not only a vital prognostic factor of BC, but also a crucial therapy targeting (20). Xu et al. have shown that MSLN is involved in various pathways, thus causing poor prognosis for ovarian cancer (OC) patients (21). The up-regulation of PDK4 was correlated with growth, proliferation, adverse outcomes and chemotherapy resistance of OC (22). The SERPINE1 could affect the expression levels of IL-6 and VEGF, thereby ultimately influencing the migration and invasion of GC (23). The study has demonstrated that KPNA2 could regulate STMN1 by E2F1 and TFDP1, which has prognostic and functional meaning (24). Wei et al. indicated that TRAF2 regulated TRAIL-induced cell apoptosis through the c-Flip/Caspase-8 pathway, suggesting that TRAF2 might be a neoteric biomarker for predicting outcome in prostate cancer (PC) patients (25).

The tumor microenvironment (TME) included various cells of the immune system, interstitial cells, extracellular matrix and tumor blood vessels (26, 27). Study have shown that immunosuppression and angiogenesis often occurred together. This could affect TEM and regulate the occurrence and progression of tumor. Combining immunotherapy with anti-angiogenic therapy may reverse the balance of the TEM (28). The infiltrating immune cell levels in TME usually change with tumor progression. In our study, patients with higher risk scores had higher ImuneScores, StromalScores and ESTIMATEScores. The CIBERSORT analysis revealed that T cells follicular helper, Macrophages M1, Monocytes, Dendritic cells resting and Macrophages M2 were significantly different between low and high risk patients. In addition, ssGSEA analysis indicated that the levels of CD8+ T cells, B cells, DCs, Macrophages, Mast cells, Neutrophils, T helper cells, TIL and Treg were less in the low risk patients than in the high risk patients. These results suggested that the aberrant immune cells infiltration might be relevant with GC progression. Particularly, the infiltration levels of immunosuppressive Treg and macrophages M2 in the low-risk class were lower than those in the high-risk class. It was suggested that the poor prognosis of GC patients in the high risk group might be associated with the immunodepression microenvironment.

Immunotherapy and chemotherapy combined with surgery have turned into the main treatment methods for GC. Given the consequence of ICP in immunotherapy, we evaluated the sensitivity of GC patients to immunotherapy by detecting differences in immune checkpoints between low and high risk patients (29). We found that a majority of the ICP genes were down-regulated in the low risk patients. Moreover, studies have indicated that the TMB and MSI were relevant with immune and targeted therapies of cancer (30, 31). In this research, we discovered that the TMB and MSI scores in the low-risk patients were higher than those in the high-risk patients, and the prognosis of patients with high TMB and MSI scores was worse, which might be related to immune effects. The TIDE score was widely applied to forecast immunotherapy sensitivity in many tumor patients (32). The TIDE contains two potential tumor immunologic evasion mechanisms: T-cell exclusion and T-cell dysfunction. The TIDE, T-cell dysfunction and T-cell exclusion score of patients in the high risk patients were significantly higher than those in the low risk patients, suggesting that low risk score patients might be more sensitive to immunotherapies. Previous research have shown that IPS could be applied to forecast the immunotherapy response of tumor patients (33). The IPS of GC patients with low risk scores were higher than those patients with high risk scores, which indicated that patients in the high risk group might possess the poor immunotherapy response.

Although the ARGs signature we constructed showed a strong performance in forecasting the outcome of GC patients, this study still had limitation. All GC samples used in this study came from public databases. It was not further verified by our own clinical specimens. We will further recruit a wide range of clinical patients in the future. GC tissue samples will extract from patients. The expression of model-associated genes in GC tissue samples will be detected. At the same time, we will combine the clinical information of patients to further verify the clinical value of our model.

## Conclusion

In conclusion, we developed an original ARGs signature to forecast the survival outcomes of GC patients. We also evaluated the immune status and immunotherapy response of different GC patients. The combination of anoikis and prognosis of patients provided a new idea for the subsequent tumor-related research. At the same time, this research expanded the understanding of the characteristics of TME. We hope to provide a new perspective for the individualized treatment of GC patients.

## Data availability statement

The original contributions presented in the study are included in the article/**Supplementary Material**. Further inquiries can be directed to the corresponding authors.

## Author contributions

YG, QM, and GO designed the study. YG analyzed the data. JC drafted the manuscript. KH and YG revised the manuscript. KC, YM, and YD contributed to the writing of the manuscript. All authors read and approved the final article.
